# The effect of integrating knowledge‐based planning with multicriteria optimization in treatment planning for prostate SBRT

**DOI:** 10.1002/acm2.13940

**Published:** 2023-02-24

**Authors:** Sandun Jayarathna, Xinglei Shen, Ronald C. Chen, H. Harold Li, Kenny Guida

**Affiliations:** ^1^ Department of Radiation Oncology University of Kansas Cancer Center Kansas City KS USA; ^2^ Department of Radiation Physics The University of Texas MD Anderson Cancer Center Houston TX USA

**Keywords:** knowledge‐based planning, multi‐criteria optimization, prostate SBRT, RapidPlan, rectal toxicity

## Abstract

Knowledge‐based planning (KBP) and multicriteria optimization (MCO) are two powerful tools to assist treatment planners in achieving optimal target coverage and organ‐at‐risk (OAR) sparing. The purpose of this work is to investigate if integrating MCO with conventional KBP can further improve treatment plan quality for prostate cancer stereotactic body radiation therapy (SBRT). A two‐phase study was designed to investigate the impact of MCO and KBP in prostate SBRT treatment planning. The first phase involved the creation of a KBP model based on thirty clinical SBRT plans, generated by manual optimization (KBP_M). A ten‐patient validation cohort was used to compare manual, MCO, and KBP_M optimization techniques. The next phase involved replanning the original model cohort with additional tradeoff optimization via MCO to create a second model, KBP_MCO. Plans were then generated using linear integration (KBP_M+MCO), non‐linear integration (KBP_MCO), and a combination of integration methods (KBP_MCO+MCO). All plans were analyzed for planning target volume (PTV) coverage, OAR constraints, and plan quality metrics. Comparisons were generated to evaluate plan and model quality. Phase 1 highlighted the necessity of KBP and MCO in treatment planning, as both optimization methods improved plan quality metrics (Conformity and Heterogeneity Indices) and reduced mean rectal dose by 2 Gy, as compared to manual planning. Integrating MCO with KBP did not further improve plan quality, as little significance was seen over KBP or MCO alone. Principal component score (PCS) fitting showed KBP_MCO improved bladder and rectum estimated and modeled dose correlation by 5% and 22%, respectively; however, model improvements did not significantly impact plan quality. KBP and MCO have shown to reduce OAR dose while maintaining desired PTV coverage in this study. Further integration of KBP and MCO did not show marked improvements in treatment plan quality while requiring increased time in model generation and optimization time.

## 1 INTRODUCTION

Stereotactic body radiation therapy (SBRT) has risen in popularity for treatment of early‐stage prostate cancer over the last decade. Typically, SBRT is delivered over five treatment fractions, delivering a total dose of 35–40 Gy to the planning target volume (PTV) while minimizing dose to nearby organs‐at‐risk (OARs).^1^, ^2^, ^3^ Due to large doses per fraction, it is important to reduce dose to normal tissues, including bladder and rectum.^4^, ^5^ Several strategies have been utilized to reduce rectal dose, including hydrogel spacer injections, which increases the distance between the prostate and rectum,^6^ and the implementation of MRI‐guided adaptive SBRT.^7^, ^8^ Even with the use of rectal spacer, the role of treatment planning is essential in delivering prescription dose to the prostate while meeting normal tissue tolerances. Advances in treatment planning over the past decade have enabled the radiation oncology community to reap the benefits of faster optimization and calculation times, automated planning processes, and higher accuracy in dose calculation.^9^, ^10^, ^11^, ^12^ Techniques that have long been researched have made their way into clinical implementation by way of commercial software. Knowledge‐based planning (KBP) and multicriteria optimization (MCO) are two recent advancements in inverse planning that have become commercially available through multiple vendors.

KBP typically involves the generation of models that predict achievable OAR dose‐volume histograms (DVHs) based on a patient's anatomy.^13^, ^14^ OAR DVH data can be inferred based on distance to target volumes and the amount of overlap with target structures. Models can be generated from a library of similar patient plans; the knowledge extracted from a cohort of plans can be applied to future cases, resulting in a more automated and standardized planning approach, essentially creating a quality assurance benchmark for all future planning.^13^, ^15^ Plans added to a model library should reflect the quality of those used in the clinical setting. Eclipse (Varian Medical Systems, Palo Alto, USA) treatment planning system (TPS) provides a KBP engine in the form of RapidPlan (RP), which allow users to train models and deploy them for routine clinical practice after proper validation. RP estimates OAR DVHs based on models trained via principal component analysis (PCA) and stepwise regression methods. Specifically, the OAR is divided into multiple regions: in‐field, out‐of‐field, leaf transmission, and overlap (with PTV) region with respect to the incident beams.^16^ The contribution for the final DVH from each region is modeled separately, and PCA is used to convert the DVHs into principal component scores (PCSs) to facilitate the regression analysis and identify outliers within the dataset.^17^

MCO was introduced as a means of increasing efficiency in inverse planning. Rather than identifying a single solution during the optimization process, as is the case with traditional inverse planning, MCO achieves a vectorized formulation on a compromised solution based on tradeoffs along a Pareto surface.^18^, ^19^, ^20^, ^21^ Eclipse TPS introduced an MCO tool in v15.5, allowing planners to explore trade‐off optimization. The optimizer will aim to find the Pareto Front and provide plans with tradeoff information based on selected objectives. One of the enhancements made to the optimization process through MCO is the ability to optimize for homogenous dose within the PTV, thus allowing users to reduce hotspots and increase minimum dose coverage.

In prostate cancer treatment planning, both KBP and MCO have shown to provide advantages over manual optimization. The KBP method has been shown to be advantageous for pelvic IMRT planning.^22^, ^23^, ^24^, ^25^ In a retrospective analysis of patients enrolled on RTOG 126 for localized prostate cancer, researchers found that over 42% of patients in the IMRT cohort were exposed to a 5% risk of rectal toxicity, with almost 10% being exposed to a 10% excessive risk.^26^ Furthermore, Moore et al. showed that bladder and rectal doses could be reduced by replanning in accordance with constraints generated from KBP modeling.^26^ In a comparison study between manual optimization and MCO for prostate IMRT plans,^18^ PTV coverage remained the same between both cohorts, whereas MCO greatly reduced anterior rectal V_40Gy_ and V_60Gy_ (69.9% to 48.6% and 35.3 to 19.4%, respectively) as well as D_1%_ for all nearby OARs; in a blinded review arm of this study, physicians also rated the MCO plans higher, on average.

The challenges of SBRT planning for prostate cancer are even higher than conventional fractionation, as excessive dose could lead to increased risk of fistula for both the rectum and bladder. In a recent HyTEC publication for prostate SBRT, the authors found that high rectal dose correlated with an increase in severity for rectal toxicity.^5^ In a dose escalation study to 50 Gy in 5 fractions, a V_50Gy_ of 3 cc or more and V_39Gy_ of 35% were underlying factors in patients with grade 3–4 rectal toxicity. Researchers at Sunnybrook Hospital noted that almost 20% of patients had grade 2 or worse rectal toxicity post‐SBRT and noticed a correlation between V_38Gy_ > 2% with late side effects.^27^

Both KBP and MCO represent enhancements to the inverse planning process, yet there is uncertainty in which tool is more clinically viable. Furthermore, commercial KBP and MCO tools present a budget issue, and clinicians may be limited to adding only one of these to their arsenal. Our goal with this study was to determine if clinics would benefit most from KBP, MCO, or the combination of both optimization tools. To answer this question, we designed a two‐phase investigation: Phase 1 explores plan comparisons between manual optimization, MCO, and KBP, and Phase 2 investigates the impact of integrating MCO with KBP on plan quality for prostate SBRT, through either “non‐linear integration” (plans utilizing a KBP model constructed from a database of MCO plans) or “linear integration” (plans utilizing additional MCO optimization after KBP model, based on manual plans, was employed). A set of ten validation plans were used to compare both KBP models against clinical plans and MCO‐only optimized plans. Dosimetric data was captured from each validation plan to make comparisons and identify the optimal planning method for prostate SBRT. In this investigation, we aim to evaluate the variability of planning outcomes, OAR doses, plan quality using MCO, KBP, or a combination of both.

## 2 MATERIALS AND METHODS

### 2.1 Patient selection and clinical treatment planning

A cohort of 40 patients previously treated with SBRT for early‐stage prostate cancer was selected from our clinical database. Thirty patient datasets were randomly selected for KBP modeling, with the remaining ten set aside for validation testing. SBRT plans were designed to deliver 3625 cGy in five fractions and all plans normalized to achieve a V_100%_ of 98%. Treatment planning was performed with Varian's Eclipse v15.6, utilizing 2–3 VMAT arcs on a TrueBeam linear accelerator (Varian Medical Systems, Palo Alto, USA) with 6 or 10 MV (with flattening filter) photons, depending on patient size; 10MV photon energy was utilized for patients with an average depth of 15 cm or greater relative to isocenter. Full arcs were utilized with 20‐degree collimator rotations used for arcs; for cases utilizing a third arc, a 90‐degree collimator rotation was made. All clinical plans were optimized with Eclipse's Photon Optimizer (PO). Departmental planning goals are listed in Table 1.

**TABLE 1:** Dose‐volume histogram (DVH) constraints for PTV and organ‐at‐risk (OARs) based on departmental standards

Target/OAR	Clinical constraints
PTV	V_100%_ ≥ 98–95%
PTV	D_99%_ ≥ 95–93%
PTV	D_0.03cc_ ≤ 107–110%
Rectum	V_3600cGy_ ≤ 1–3 cc
Rectum	V_3260cGy_ ≤ 10–15%
Rectum	V_2900cGy_ ≤ 20–25%
Rectum	V_1810cGy_ ≤ 50–55%
Bladder	V_3700cGy_ ≤ 10–20 cc
Bladder	V_1810cGy_ ≤ 40–45%
Femur Head L	V_1450cGy_ ≤ 5–10%
Femur Head R	V_1450cGy_ ≤ 5–10%

*Note*: Prostate stereotactic body radiation therapy (SBRT) planning goals and constraints.

Abbreviation: PTV, planning target volume.

### 2.2 Phase 1: KBP model configuration and validation (Manual vs. MCO vs. KBP_M)

The KBP_M model was created by extracting plan data, including DVHs, structure sets, and beam geometry, from the manually optimized cohort. Once the required data were extracted, training commenced for model configuration. The model configuration module in Varian ARIA (Varian Medical Systems, Palo Alto, USA) provides statistical analysis to showcase the training results, where *R* ^2^ and *χ* ^2^ provide a firsthand estimation of the quality of training. Furthermore, statistics on structures matched, in‐field DVH data, and outlier plans can be utilized to further improve the model. Cook's distance (CD) and modified Z‐score (mZ) were two statistical tools used to identify possible geometric and dosimetric outliers; the RP model configuration module performed statistical analyses and outlined plan structures with CD > 10 or mZ > 3.5, allowing us to examine these cases more closely to determine if further planning was needed to improve the model. Specifically, CD provides a measure of regression fit parameters for any outliers by omitting the plans from the regression analysis, while mZ is more robust compared to the standard Z‐score since it considers the median when calculating the Z‐scores.^16^, ^28^ Dosimetric outliers were replanned and reimported into the models and training was performed again to determine the significance of improvement. If replanning did not further impact the model, the plan was labeled as a geometric outlier. If removing the geometric outlier did not improve the model, then the plan remained in the model. After finalizing model training, we were left with the finished model, termed KBP_M, based solely on manually optimized plans.

**FIGURE 1:**
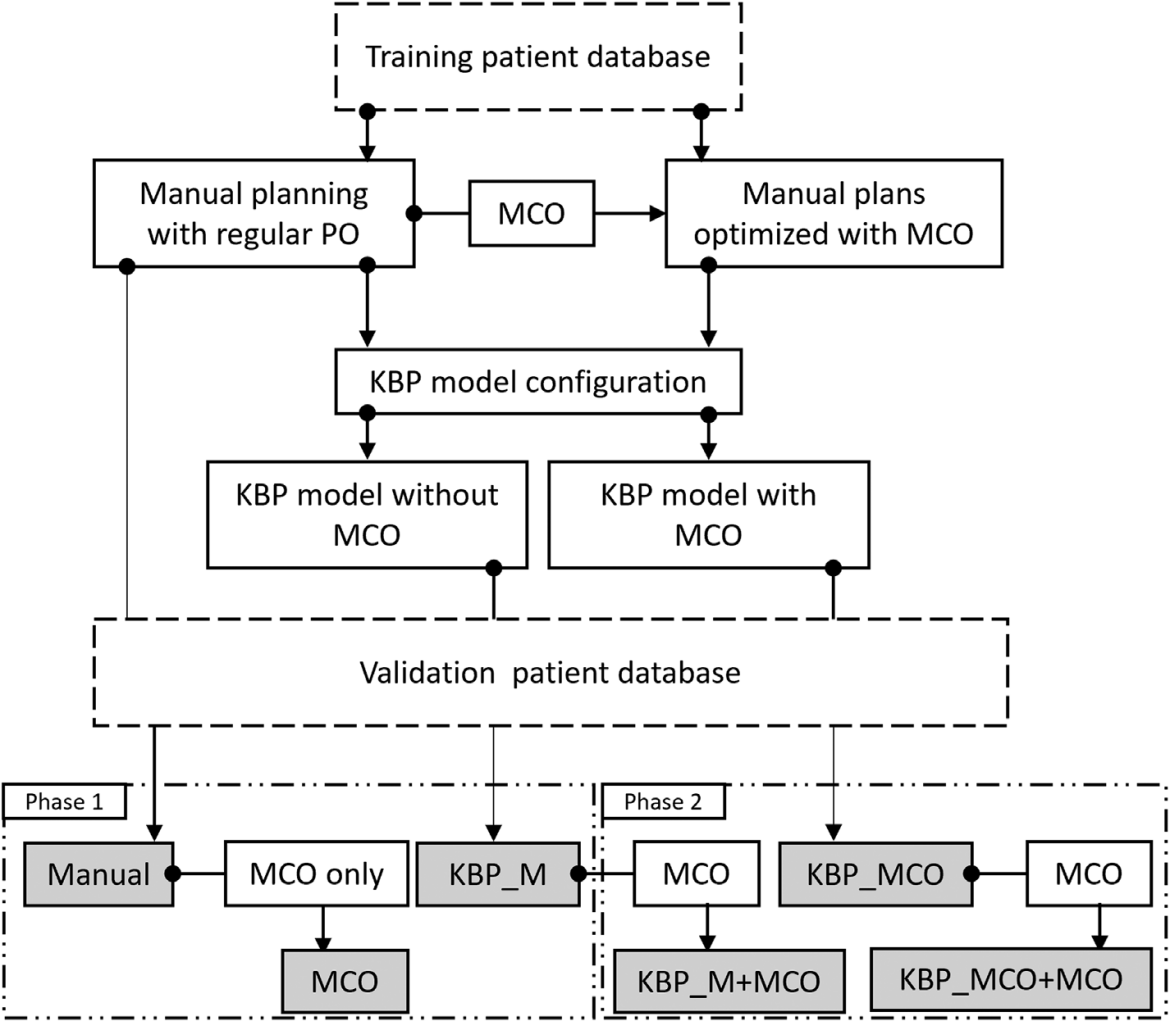
The flow of planning and model configuration for the training and validation. For this analysis two KBP models and six independent plans for each validation patient were created and compared for dosimetric details. Here in Phase 1: validating any clinical significance of plans optimized with MCO only and validating clinical significance of plans optimized with KBP. In Phase 2: investigating different methodologies of integrating MCO into KBP and then to investigate any phan quality enhancement. KBP, knowledge‐based planning; MCO, multicriteria optimization.

In the first phase, three different plans were generated for each patient in the validation set, as shown in Figure 1: Manual, MCO, KBP_M. Manually optimized plans were treatment approved by radiation oncologists in our department. Next, these plans were copied and further optimized with tradeoff optimization to create a set of MCO plans. Finally, the KBP_M model was applied to each patient in the validation set and a knowledge‐based plan was optimized for each case.

### 2.3 Phase 2: MCO integration: Model configuration and validation (KBP_MCO, KBP_MCO+MCO, KBP_M+MCO)

In the second phase of our initiative, the original model cohort was copied, and plans were further optimized with MCO to create a second dataset for KBP modeling. Again, these plans were analyzed to ensure departmental constraints were achieved upon plan finalization. This MCO cohort was uploaded into a new model in RP and statistically analyzed by the software. Plans that were flagged were re‐optimized to determine if they were dosimetric or geometric outliers. Once the KBP_MCO model was finalized, the second phase of validation tests began.

With the completion of the second model, another set of three independent plans were generated for each patient in the validation cohort, as shown in Figure 1: KBP_M+MCO (linear integration), KBP_MCO (non‐linear integration), and KBP_MCO+MCO (a combination of both integration methodologies). The aim of this phase was to investigate different methods of integrating MCO into KBP. KBP_M plans from the first phase were further optimized to generate KBP_M+MCO plans (linear integration). Next, plans were generated with the KBP_MCO model for each patient in the validation set (non‐linear integration). Finally, these plans were further optimized with MCO to create KBP_MCO+MCO plans (mixed integration).

Thus, six different planning techniques were introduced and compared throughout this analysis, namely:

1. Manual: Plans created without advanced planning techniques,
2. MCO: Manual plans optimized with additional MCO,
3. KBP_M: Plans generated via a KBP model trained with manual plans,
4. KBP_M+MCO: KBP_M plans optimized with additional MCO,
5. KBP_MCO: Plans generated via a KBP model trained with MCO plans, and
6. KBP_MCO+MCO: KBP_MCO plans optimized with additional MCO.

### 2.4 Planning analysis

Finally, all plans were analyzed using ClearCheck (RadFormation, New York, NY) via ESAPI script to assess plan quality. An in‐house constraint set was configured for use in ClearCheck to thoroughly analyze plan quality; along with PTV coverage and OAR constraints, as shown in Table 1, conformity index (CI), heterogeneity index (HI), and gradient index (GI) were extracted for cross comparison among different planning techniques by using this ESAPI script. Throughout the analysis, we used the following definitions for quality indices.^29^

$$CI_{100}=\frac{V_{100\%}}{\mathrm{TV}}$$

where V_100%_ is the prescription isodose volume and TV target volume,

$$\mathrm{HI}=\frac{D_{2\%}-D_{98\%}}{D_{50\%}}$$

where D_2%_ is the dose to 2% of the PTV, D_98%_ the dose to 98% of the PTV, and D_50%_ the dose to 50% of the structure volume, and

$$\mathrm{GI}=\frac{R_{50\%}}{R_{100\%}}$$

where R_50%_ is the radius (in cm) of an equivalent sphere with the same volume as the 50% isodose volume, and R_100%_ the radius (in cm) of an equivalent sphere with the same volume as the 100% isodose volume.

Beyond plan quality, further examination was performed on the two KBP models. Plan optimization times were captured for patients in the validation cohort to determine the effect of KBP and MCO on planning efficiency. Statistical analysis data from RP was captured for both KBP_M and KBP_MCO. Model‐based predictions for departmental OAR constraints were captured at the time of optimization using each model for each validation patient in order to determine the quality and performance of each model. Specifically, statistical analysis data, including V_3600cGy_, V_3260cGy_, V_2900cGy_, V_1810cGy_, and mean dose for the rectum, V_3700cGy_, V_1810cGy_, and mean dose for the bladder, and mean dose for the femoral heads, were collected at two different levels. The first level data were collected prior to optimization from the derived DVH predictions for each validation patient, which were extracted from the DVH line constraints in the optimizer window.^30^, ^31^, ^32^ The second level data were collected from the plan DVH after the optimization and dose calculation were completed. Paired *t*‐tests were carried out between manual and validation plans, with a focus on DVH metrics for the rectum and bladder. A Wilcoxon signed‐rank test was carried out between manually created and validation plans for each patient, with a focus on DVH metrics for the rectum and bladder. A case study was provided for one of the ten validation patients to demonstrate model prediction capabilities as well as optimization performance.

## 3 RESULTS

### 3.1 Phase 1: Manual vs. MCO vs. KBP_M

DVH data were averaged across each validation plan type for dosimetric comparisons, as seen in Table 2. All plans in the validation set ensured the PTV V_100%_ goal of 98% was achieved. Compared to manual optimization, MCO and KBP_M reduced rectal dose by at least 2 Gy, while reducing rectal V_1810cGy_ by 41.4% and 36.5%, respectively. Both MCO and KBP_M improved PTV homogeneity, from 0.083 to 0.034 and to 0.046 (improvements of 59% and 45%), respectively. There were no significant differences in bladder dose metrics between the three optimization strategies. Of the three planning techniques, KBP_M achieved the lowest CI_100_, on average.

**TABLE 2:** Achieved organ‐at‐risk (OAR) doses and standard deviations across all planning methods. The plan quality indices CI_100_, HI, and GI are shown for the PTV

Metric	Manual	MCO	KBP_M	KBP_M+MCO	KBP_MCO	KBP_MCO+MCO
Rectum						
V_3600cGy_ (cc)	0.81 ± 0.25	0.86 ± 0.32	0.89 ± 0.36	0.99 ± 0.355	0.80 ± 0.30	0.81 ± 0.35
V_3260cGy_ (cc)	3.46 ± 0.86	3.23 ± 0.75	3.05 ± 0.89	3.02 ± 0.796	2.79 ± 0.64	2.83 ± 0.73
V_2900cGy_ (cc)	6.60 ± 1.26	5.32 ± 1.02	5.28 ± 1.01	5.06 ± 1.02	5.11 ± 0.76	4.88 ± 0.90
V_1810cGy_ (cc)	26.78 ± 4.52	15.68 ± 2.22	17.00 ± 1.54	15.82 ± 1.44	17 ± 1.60	16.34 ± 1.13
Mean (cGy)	1165 ± 110	948 ± 68	966 ± 42	917 ± 50	979 ± 39	943 ± 42
Bladder						
V_3700cGy_ (cc)	4.11 ± 0.69	2.60 ± 0.59	4.33 ± 0.75	3.24 ± 0.41	4.07 ± 0.65	3.32 ± 0.68
V_1810cGy_ (cc)	13.71 ± 2.32	13.90 ± 2.36	13.95 ± 2.42	13.75 ± 2.48	13.48 ± 2.25	13.19 ± 2.30
Mean (cGy)	731 ± 99	744 ± 103	735 ± 106	728 ± 107	717 ± 98	750 ± 99
Femur R						
Mean (cGy)	639 ± 46	686 ± 75	734 ± 54	697 ± 49	727 ± 59	656 ± 43
Femur L						
Mean (cGy)	639 ± 43	711 ± 70	747 ± 46	660 ± 56	704 ± 64	661 ± 34
PTV						
CI_100_	1.076 ± 0.01	1.074 ± 0.01	1.057 ± 0.009	1.054 ± 0.009	1.046 ± 0.009	1.049 ± 0.005
HI	0.083 ± 0.04	0.034 ± 0.00	0.046 ± 0.001	0.041 ± 0.009	0.045 ± 0.002	0.039 ± 0.001
GI	1.579 ± 0.52	1.644 ± 0.077	1.584 ± 0.053	1.623 ± 0.056	1.567 ± 0.059	1.615 ± 0.067

*Note*: Plan quality results for all validation plans.

Abbreviations: KBP, knowledge‐based planning; MCO, multicriteria optimization; PTV, planning target volume.

### 3.2 Phase 2: Linear and non‐linear integration methods

The results from Phase 2 did not indicate overwhelming improvements on plan quality metrics with either non‐linear or linear integration of KBP and MCO. Specifically, compared to the manual planning (1165 cGy), the mean rectum dose was reduced by nearly 2 Gy when a combination of MCO and KBP were applied; similar reductions in rectal V_1810cGy_ were seen for KBP_M+MCO, KBP_MCO, and KBP_MCO+MCO (41%, 37%, and 39%, respectively). V_2900cGy_ and V_3260cGy_ were improved by using all three Phase 2 techniques. While all three Phase 2 techniques improved CI_100_ by at least 2% and HI by a minimum of 46%, improvements on reducing OAR dose remained subdued; again, bladder dose metrics were mostly unchanged, while femoral head dose was on par with manual optimization. Improvements in in HI and CI_100_ were on par with MCO and KBP alone.

### 3.3 Planning analysis: MCO, KBP, or integrating KBP and MCO

**FIGURE 2:**
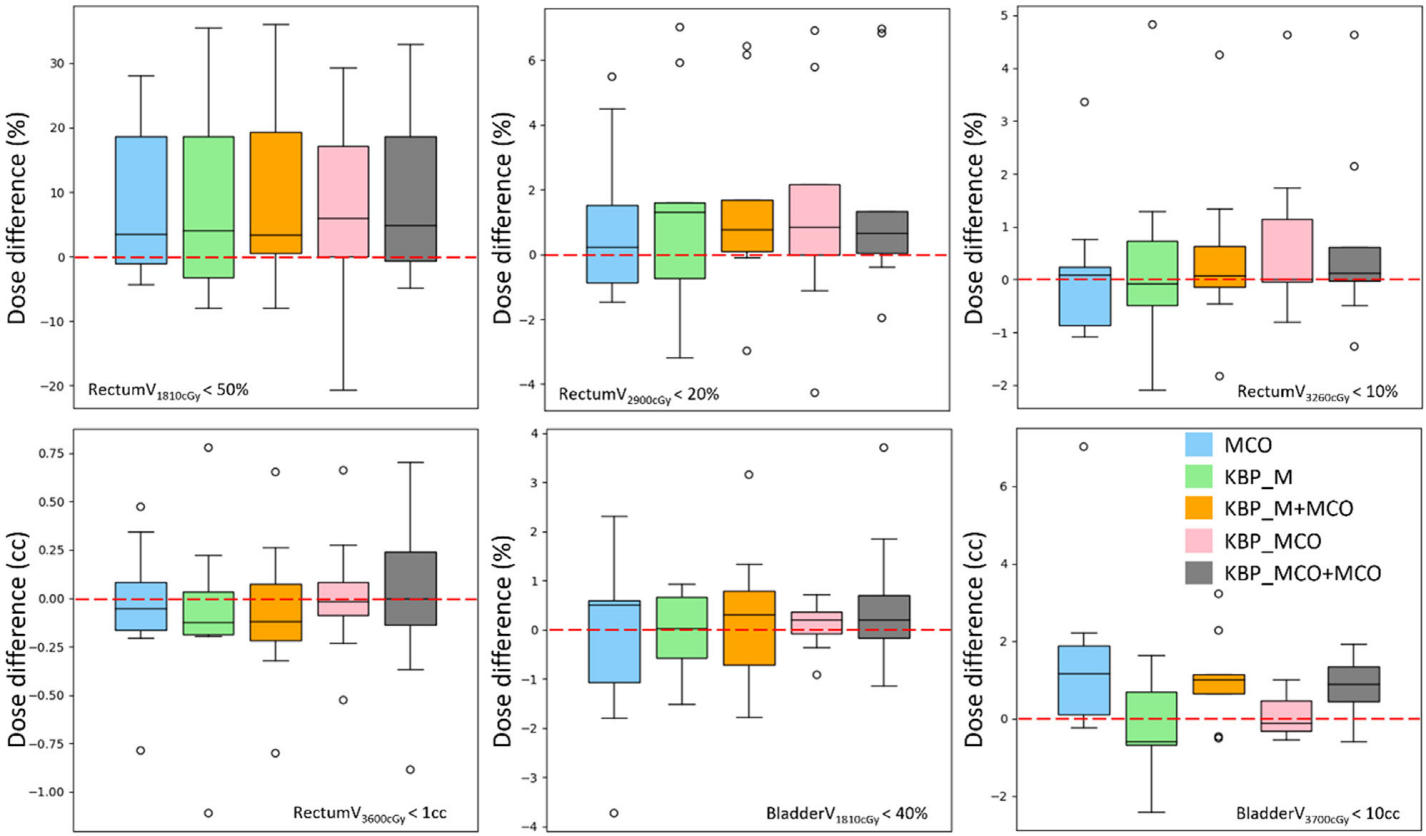
The box and whisker plots for the difference between validation and clinical plans for rectum and bladder. The midline of the box shows the median value of each dose difference. The dashed line represents the zero cutoff where, if the dose difference >0, the planning methods spared the organ‐at‐risk (OAR) doses more than the original clinical plans. KBP, knowledge‐based planning; MCO, multicriteria optimization.

With KBP, MCO, and various integration techniques improving upon the dosimetric statistics achieved by manual optimization, our next step was to investigate if one of the advanced optimization techniques separated itself from the others. To visualize differences between MCO and KBP plans from the original clinical plans, box and whisker plots were generated for clinical dose constraints, as shown in Figure 2. The y‐axis represents the difference between dosimetric indices achieved by clinical and validation plans. If we achieve better OAR sparing with the validation plans, the y‐axis value should be greater than zero (validation plan minus clinical Plan). An immediate improvement is shown among all the techniques investigated. Specifically, MCO and KBP_M both show similar results, suggesting either MCO optimization or KBP modeling can improve dosimetric constraints. For both bladder and rectum, the similar mean positive deviations were seen in the KBP_M+MCO, KBP_MCO, and KBP_MCO+MCO data sets, which suggest that integrating MCO linearly, non‐linearly, or as a combination result in a similar outcome. Overall, most of the planning techniques are above the zero line; implementing MCO, KBP, or some combination of both reduced OAR DVH metrics better than manual optimization for most of the validation cases.

KBP_MCO+MCO planning was the only technique to significantly reduce bladder dose (*p*‐value < 0.05), as seen in Table 3. All advanced optimization techniques significantly improved rectal dose metrics, as compared to manual optimization. Quantitatively, MCO, KBP, or MCO+KBP reduced rectum V_3260cGy_ by 13%, V_2900cGy_ by 22%, and V_1810cGy_ by 38%, as compared to manual optimization. This finding suggests that using KBP or MCO can significantly improve plan quality, in terms of rectal dose sparing, for prostate SBRT plans. While the KBP_MCO+MCO planning seems to provide high‐quality plans across the validation set, the results are not significantly different from MCO or KBP optimized plans, other than a greater reduction in bladder dose V_3700cGy_, by 19%.

**TABLE 3:** Paired *t*‐test results for rectum and bladder, for each validation plan type versus the original, manually optimized plans. Only *p*‐values <0.05 are considered significant. Multicriteria optimization (MCO) only, MCO linear integration, MCO non‐linear integration and a combination of MCO integration all have significant contribution for rectal dose reduction. For bladder, only a combination of MCO integration shows a significant dose reduction.

OAR	MCO	KBP_M	KBP_M+MCO	KBP_MCO	KBP_MCO+MCO
Rectum	**0.0144**	**0.0394**	**0.0161**	**0.0368**	**0.0126**
Bladder	0.1705	0.3294	0.2239	0.2893	**0.0156**

*Note*: Calculated *p*‐values for validation plans compared to manual optimization.

Abbreviations: KBP, knowledge‐based planning; MCO, multicriteria optimization; OAR, organ‐at‐risk.

Significant values in bold.

Plan optimization time was captured for patients in the validation cohort. With manual optimization, optimal plans were generated in 41 min, on average. KBP_M and KBP_MCO models reduced optimization time to 9.2 min on average (range 7–12 min). MCO optimization alone averaged 28 min from start to finish (range 25–35 min), as a database of plans was generated during this optimization process.

### 3.4 KBP modeling improvement due to MCO optimized plans

**FIGURE 3:**
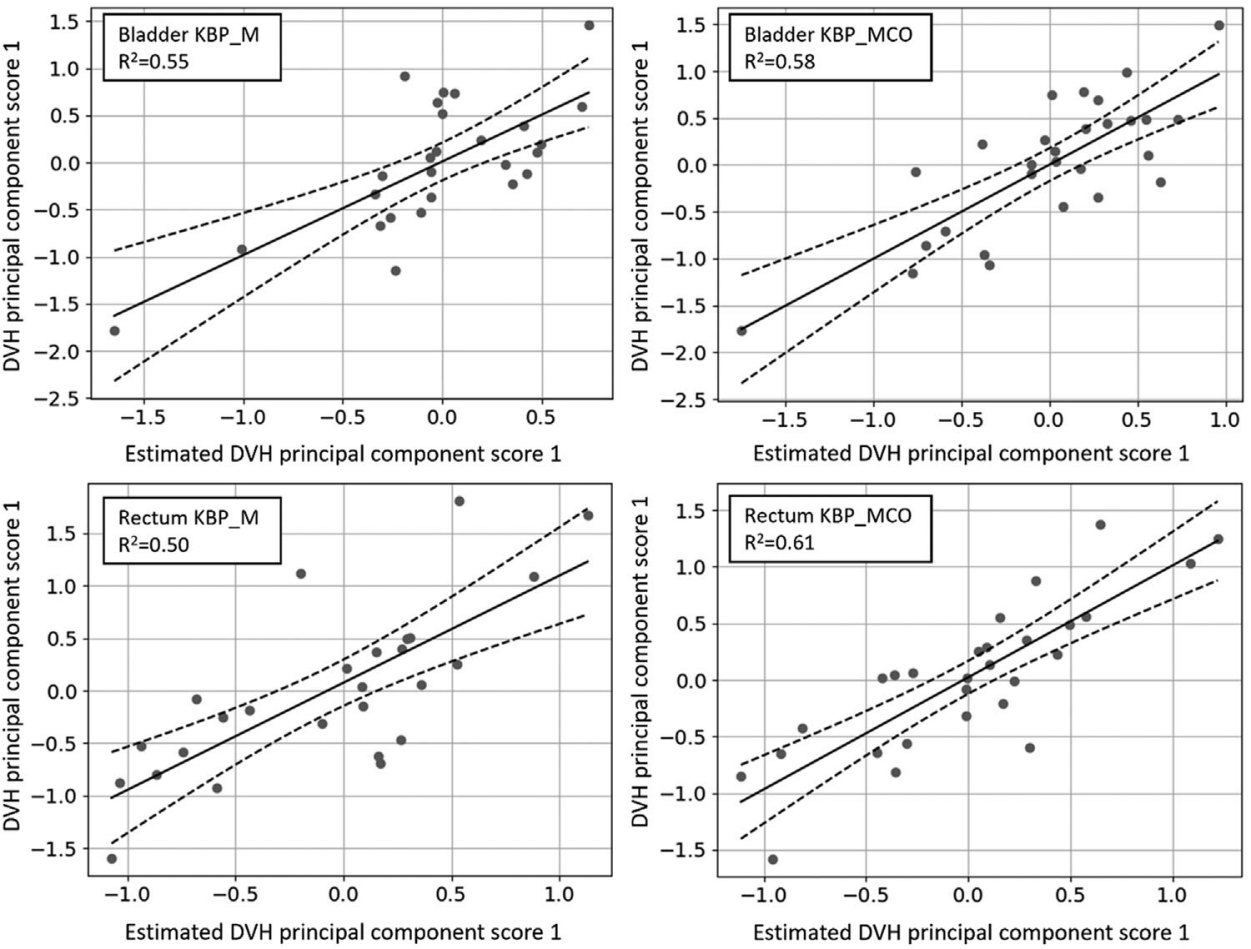
The comparison of KBP_M versus KBP_MCO for rectum (top panel) and bladder (bottom panel). The y‐axis represents DVH PCS from original data and x‐axis shows the estimated PCS. The regression line shown in solid and ± 95% significance interval is showing in dashed line. DVH, dose‐volume histogram; KBP, knowledge‐based planning; MCO, multicriteria optimization.

During KBP model configuration, the advantages of non‐linear integrated MCO optimization became apparent. Out of the 30 model plans generated for both models, only two were removed from the KBP_MCO model, whereas four plans were removed from the KBP_M model, suggesting that MCO optimization reduced the number of outlier plans in this sample set. In Figure 3, bladder and rectum regression plots (solid line) with 95% confidence level (two dashed lines) display the PCS fitting for the estimated DVH PCS versus modeled DVH PCS. *R* ^2^ values for bladder increased from 0.55 to 0.58 (5% improvement), while for rectum *R* ^2^ improved by 22%, increasing from 0.50 to 0.61, when MCO was utilized in planning. These results may partially support that non‐linear integration of MCO enhanced the quality of the model training.

### 3.5 Comparison of model predicted OAR constraints

**FIGURE 4:**
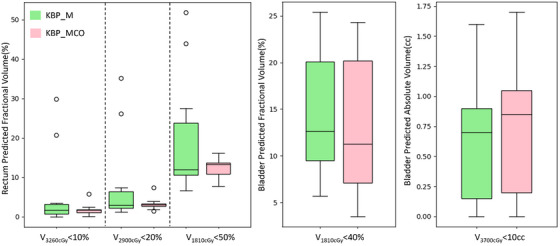
The comparison of constraint prediction of KBP_M versus KBP_MCO for rectum (left panel) and bladder (middle/right panel). The y‐axis represents predicted fractional volume or absolute volume for a given constraint. The midline of the box shows the median value and outliers are shown in circles. KBP, knowledge‐based planning; MCO, multicriteria optimization.

For both KBP_M and KBP_MCO, the predicted dose constraints were compared. The predictions were extracted from the DVH line objective, which is the lower bound of the uncertainty of the predicted DVH model for an OAR. Predicted DVH values for OARs based on departmental constraints from both models are shown in Figure 4. Model predictions show improvement for all three rectal constraints when using the KBP_MCO model, as well as a 2 Gy reduction in mean rectal dose. Furthermore, KBP_MCO reduced variations in the rectal DVH predictions across the patient set, as seen in Table 2. Specifically, V_3600cGy_ was reduced from 6 ± 4 cc to 0.2 ± 0.1 cc, V_3260cGy_ from 7 ± 3.5% to 2 ± 0.5%, V_2900cGy_ from 9 ± 4% to 3 ± 0.5%, and V_1810cGy_ from 20 ± 5% to 12 ± 1%. While the KBP_MCO model predicted that all clinical tolerances were met for all plans, KBP_M tended to overpredict rectal V_3600cGy_ (6 ± 4 cc, on average) for the validation cohort. Bladder dose predictions did not show much improvement when comparing KBP_MCO to KBP_M. Overall, predicted constraints and mean values were improved for KBP_MCO model, as seen in Table 4.

**TABLE 4:** Predicted dose‐volume histogram (DVH) constraints for the organ‐at‐risk (OARs) from two trained models. A clear improvement of the predictions is noted for the model build with non‐linear integration of multicriteria optimization (MCO). Predicted DVH values met departmental constraints except for the KBP_M rectum V_3600cGy_ (tolerance 1–3 cc)

Target/OAR	Dose Metric	KBP_M	KBP_MCO
Rectum	V_3600cGy_ (cc)	6 ± 4	0.2 ± 0.1
Rectum	V_3260cGy_ (%)	7 ± 3.5	2 ± 0.5
Rectum	V_2900cGy_ (%)	9 ± 4	3 ± 0.5
Rectum	V_1810cGy_ (%)	20 ± 5	12 ± 1
Rectum	Mean (cGy)	1027 ± 141	831 ± 40
Bladder	V_3700cGy_ (cc)	0.95 ± 0.13	0.97 ± 0.20
Bladder	V_1810cGy_ (%)	14 ± 2	13 ± 2
Bladder	Mean (cGy)	720 ± 95	681 ± 100
Femur Head L	Mean (cGy)	752 ± 79	695 ± 62
Femur Head R	Mean (cGy)	658 ± 34	564 ± 46

*Note*: KBP predicted dose metrics for selected OARs.

Abbreviations: KBP, knowledge‐based planning; MCO, multicriteria optimization; OAR, organ‐at‐risk.

### 3.6 Case study

**FIGURE 5:**
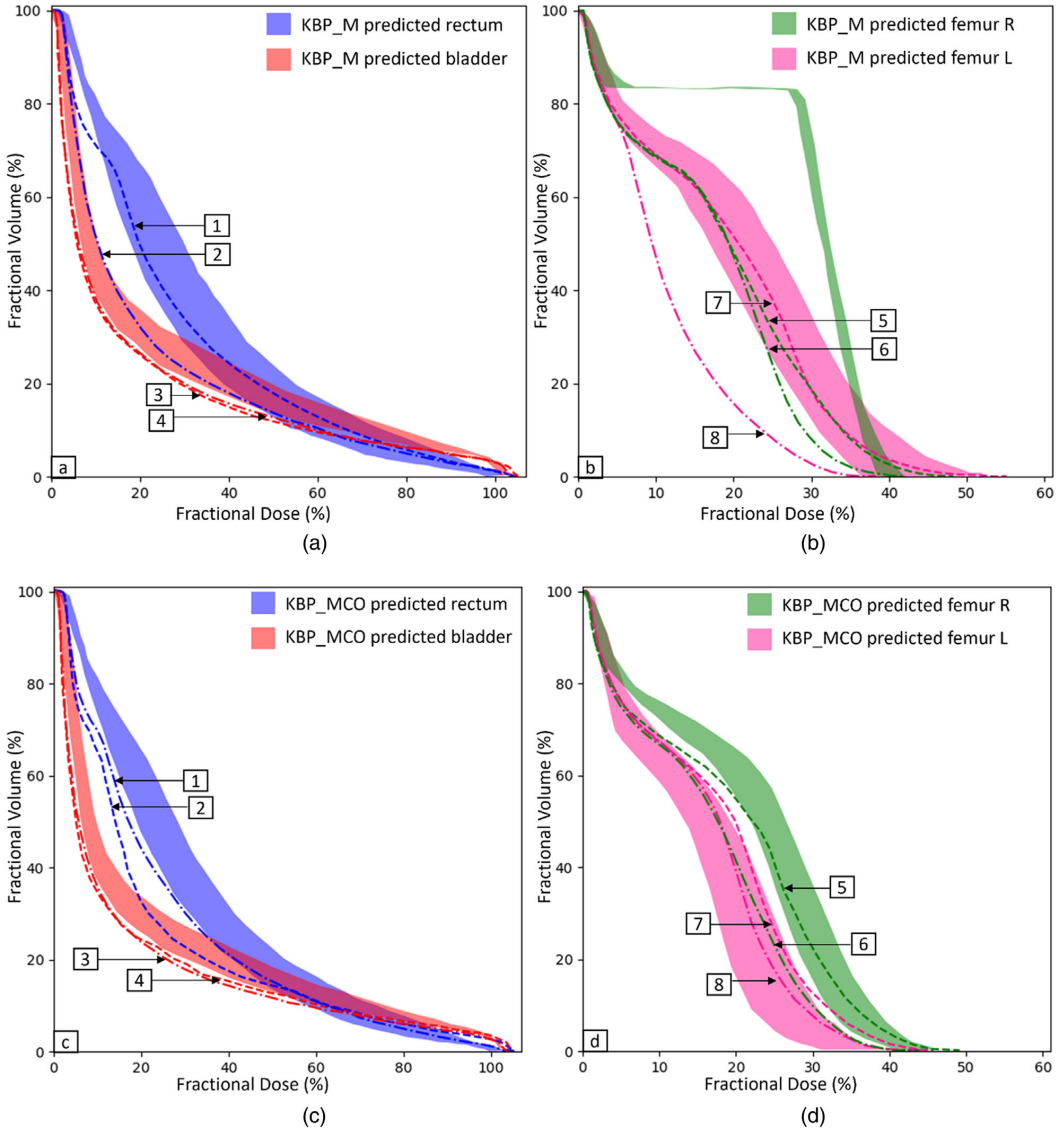
(a–d) Case study on a selected validation patient, showing predicted OAR DVH data generated by KBP_M (a, b) and KBP_MCO (c, d). The shaded area shows the predictions from the model training for each OAR and dashed lines show the achieved dose with the model (1/3 for rectum/bladder, 5/7 for femur R/L) and further MCO optimization (2/4 is for the rectum/bladder and 6/8 for the femur R/L, respectively). DVH, dose‐volume histogram; KBP, knowledge‐based planning; MCO, multicriteria optimization; OAR, organ‐at‐risk.

To further display the effects of MCO on both modeling and optimization, we selected a single patient from the validation cohort to further explore. Figure 5a–d illustrates the predicted and achieved DVHs for rectum, bladder, and both femoral heads with KBP_M and KBP_MCO, as well as achieved values for both KBP models with further MCO optimization. Predicted DVHs are depicted in color wash, showing the range for each structure. Each plot also displays the achieved OAR DVH for each model and optimization strategy. For rectum, using additional MCO optimization clearly reduces the DVH plot in the low and intermediate dose regions to a greater extent than the KBP_M model alone for this case; achieved rectal dose was also lower than the predicted range for the KBP_MCO plans as well. On the other hand, there was not a significant improvement in bladder dose with further MCO optimization; overall, the model accurately predicted bladder dose. The right femoral head did not fit the KBP_M model and the DVH was not accurately predicted, as seen by the presence of the plateau region. The left femoral head was modeled accurately, and dose was further reduced for this structure by running MCO in conjunction with KBP_M. When KBP_MCO was employed, improvements were observed in achieved and predicted doses. The predicted DVH for right femoral head improved upon the KBP_M model, likely due to higher quality modeling as MCO plans formed the basis of this model. The KBP_MCO model reduced the width of DVH prediction bands for bladder, rectum, and left femoral head in this case, displaying the reduction in plan variability in this model.

**FIGURE 6:**
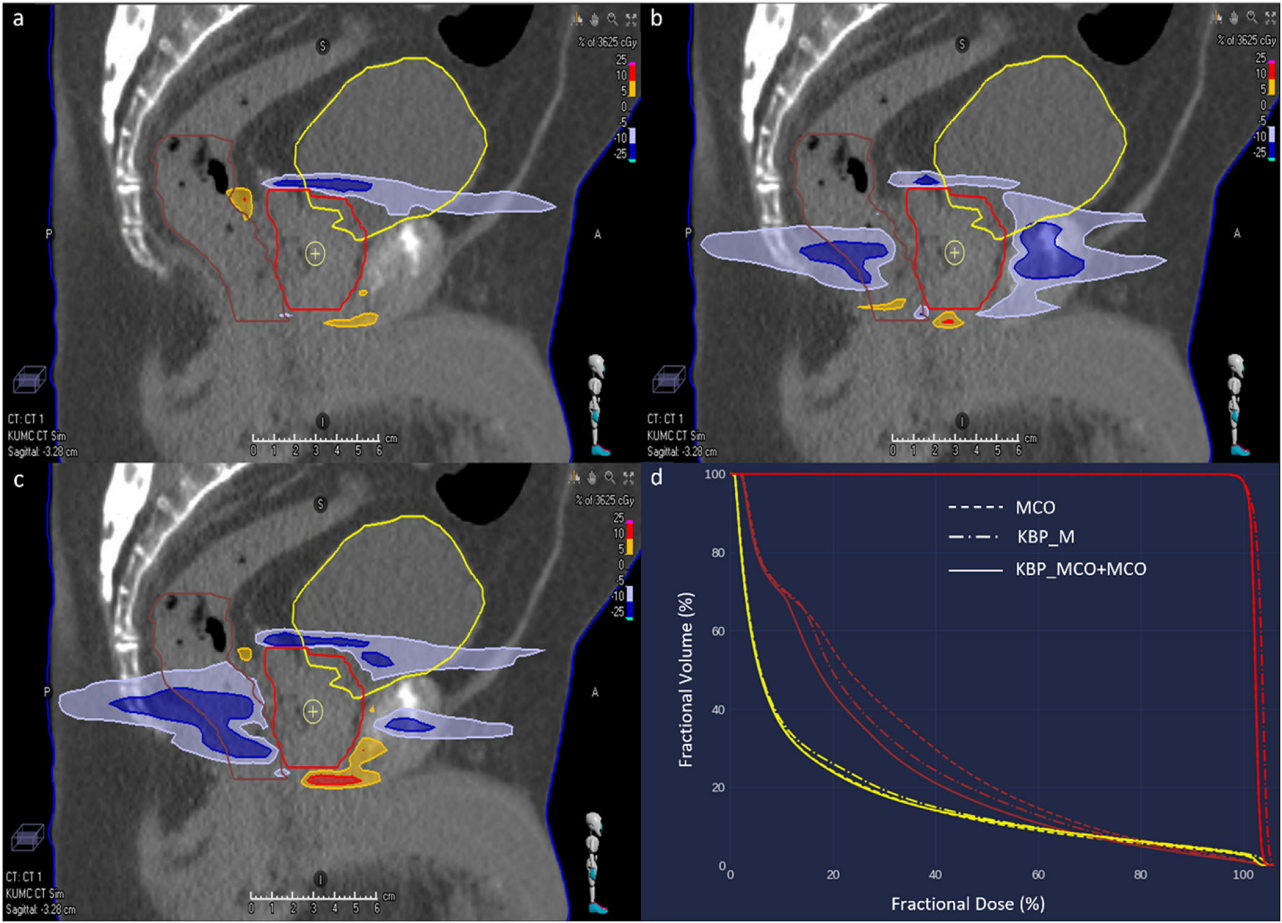
Dose subtraction sagittal views. Differences between the manual plan and MCO (a), KBP_M (b), and KBP_MCO+MCO (c). Dose reductions are shown in blue, while areas of increased dose are shown in red. A DVH (d) showing these validation plans shows improved rectal sparing with KBP‐based planning and improved PTV homogeneity with MCO. DVH, dose‐volume histogram; KBP, knowledge‐based planning; MCO, multicriteria optimization.

For the same clinical case, in conjunction with the DVH plots, dose subtraction images were generated to display the differences between MCO, KBP_M, and KBP_MCO+MCO plans with the original clinical plan, as seen in Figure 6. The dose subtraction sagittal views show the difference between the manual plans versus MCO (6a), KBP_M (6b), and KBP_MCO+MCO (6c). With MCO alone, we did not see much improvement in rectal dose, but we were able to reduce dose heterogeneities within the PTV (reduced from 0.041 to 0.032). Optimizing with KBP did reduce rectum and bladder dose but increased the dose heterogeneity in the PTV slightly (0.05), as seen in the DVH (6d). KBP_MCO+MCO further improved rectal sparing to a greater extent than KBP or MCO alone, while nearly achieving the level of dose homogeneity (0.037) as the MCO plan. KBP, MCO, and KBP_MCO+MCO all improved rectal V_1810cGy_ by at least 14%. For this case, KBP_MCO+MCO had the greatest impact on rectum V_3600cGy_ and bladder V_3700cGy_, reducing these dose metrics from 0.77 cc and 5.184 cc to 0.62 cc and 3.71 cc, respectively. This case highlights the patient specific subtle improvements achieved by the combination of non‐linear and linear integration, as all three plans represent improvements over manual optimization.

## 4 DISCUSSION

In this study, we set out to determine the roles of KBP and MCO optimization in the clinical setting, specifically for prostate SBRT treatment planning. We utilized a KBP model, based on clinical plans, and MCO on a set of patients and compared the resulting plans to manually optimized treatment plans. From our perspective, both conventional KBP and MCO‐only improved plan quality significantly over manual optimization.

We further explored integrating MCO optimization into KBP model construction and testing if the non‐linear integration method could unlock a new potential for treatment planning. While our model statistics and DVH predictions showed improvement over the KBP_M model, plan quality results did not show significant improvement with the non‐linear integration approach.

We investigated linear integration techniques, namely optimizing with the additional MCO engine after running the plans using both KBP models. Even though MCO provides additional optimization tools, our plan quality did not differ much from MCO or KBP alone. Perhaps for other body sites, where OARs and PTVs are numerous, a solution could be more apparent. Our group hopes to investigate this in the future.

Even the most experienced planners may benefit from KBP and MCO. Using either tool, we showed that we were able to reduce rectal V18Gy by over 10% and mean dose by over 2 Gy. We achieved better target conformity and improved PTV heterogeneity using KBP and MCO. Still, the question remains: how to choose between KBP versus MCO for clinical use?

KBP planning reduced treatment planning time, as we were able to achieve desired quality plan in one or two iterations. Similar studies have shown that KBP can significantly reduce planning time; Chang *et. al*. saw a reduction in nasopharynx plan times, from 295 to 64 min, when KBP was introduced into the planning process.^33^ The automation of optimization constraints and weights helped streamline the optimization process and guide planners in designing optimal plans. However, generating KBP models in a busy clinic requires a significant time investment, as model generation is a time‐consuming process. Finding patients to serve as the basis for a model, exporting DVH results, and model construction and validation take time and resources, as well as teamwork between physicists, dosimetrists, and physicians.

All MCO plans were run with a traditional MCO optimizer in this study. KBP optimization ranged from 7–10 min for patients on this study. Because of the few structures involved in prostate SBRT plans, MCO optimization time ranged from 25–35 min for patients on this study; for more complex plans, such as head and neck, we have experienced 1–1.5 h of MCO plan optimization. With a greater number of targets and OARs, MCO optimization time can be significant based on our clinical experience. Despite the optimization time associated with MCO, this technique has been found to reduce planning time, even for complex head and neck cases; Kierkels saw a reduction of 160 min when employing MCO optimization for complex head and neck IMRT cases, as compared to manual optimization.^34^ Adding additional optimization time with MCO after KBP optimization only added to the total planning time, without much added clinical benefit. In our clinical experience, there is a larger learning curve in utilizing MCO optimization when compared to incorporating KBP into the planning process. While both tools allow the users to understand what is clinically possible, based on the prescribed dose, PTV and OAR geometry, and delivery technique, there are often many decisions on dosimetric tradeoffs to select in MCO planning; determining the optimal tradeoffs for each case takes practice.

Due to the scope of this study, we limited the number of plans in the modeling and validation sets to thirty and ten, respectively. In the literature, studies have ranged from a floor of 25 patients to 75 patients needed to generate accurate DVH predictions.^35^, ^36^ We did notice that our KBP_M model tended to overestimate rectum dose predictions for V_3600cGy_ and failed to accurately model femoral heads for some cases, as seen in Figure 5b, suggesting that we may need more cases to improve the capabilities of this model. Our KBP_MCO model's dose predictions closely represented our achieved dose, displaying the strength of the model and, perhaps, indicating that fewer plans are needed for a successful model when comprised of MCO optimized plans. As our KBP program expands, we will look to continuously add more high‐quality plans to our prostate SBRT models.

Both KBP and MCO showed promise improving plan quality for prostate SBRT. As a clinic fortunate enough to have both tools, we were able to investigate the benefits of both techniques in the planning process. Our results demonstrate that MCO optimization and creating KBP models based on quality plans proved to be superior to relying simply on manual optimization alone; utilizing KBP with further MCO optimization improved our plans, but not to the extent we originally envisioned. MCO planning yielded high‐quality plans across our dataset that reduced OAR dose. Building a model based on MCO plans seemed to have a positive impact on our model quality, but again, the results did not separate themselves from the other techniques. With a dedicated physics and dosimetry group, a clinic could greatly benefit from the use of KBP models if clinical resources were appropriated. Having a large center with many similar cases would provide the radiation oncology team with the opportunity to model further plans from a selection of high‐quality treatment plans, thus greatly reducing planning time and variations for successive patients. For smaller centers without an abundance of similar, high‐quality plans, an investment in MCO could prove worthy, as MCO can be integrated directly into the treatment planning process without the needs for model generation and validation.

5

In this research study, we compared KBP and MCO to manual optimization for prostate SBRT, finding that both advanced planning techniques provided clinical advantages in plan quality. Compared to manual optimization methods, incorporating MCO, KBP or a combination of both can reduce the rectal dose in prostate SBRT plans by 2 Gy on average, which could be beneficial for prostate SBRT to reduce any rectal toxicities. While rectal dose metrics were improved by the use of MCO and KBP, bladder dose metrics did not see much improvement from the use of these two advanced optimization techniques. Both MCO and KBP plans improved HI and CI_100_ for the PTV across the validation set. Further integration of MCO and KBP did not significantly impact plan quality, even though linear integration of the two showed improvements to model metrics. Future studies will aim to determine which optimization method could be more clinically viable for other body sites.

## AUTHOR CONTRIBUTIONS

Sandun Jayarathna: Lead author, treatment planning, data collection, manuscript review, preparation, revision, and final approval. Xinglei Shen: Treatment plan review, manuscript review, and preparation. Ronald C. Chen: Treatment plan review, manuscript review, and preparation. H. Harold Li: Manuscript review, revision, and preparation. Kenny Guida: Corresponding author, treatment planning, data collection, manuscript review, preparation, revision, and final approval.

## CONFLICT OF INTEREST STATEMENT

Dr. Chen reports personal fees from Myovant, personal fees from Abbvie, personal fees from Accuray, personal fees from Astellas, personal fees from Janssen, personal fees from Bayer, outside the submitted work.
